# Preiser disease in a child before complete ossification of the scaphoid: a case report

**DOI:** 10.1186/s12891-022-05226-8

**Published:** 2022-03-18

**Authors:** Taketsugu Fujibuchi, Hiroshi Imai, Akihiro Jono, Hiroshi Kiyomatsu, Hiromasa Miura

**Affiliations:** Department of Bone and Joint Surgery, Ehime University Graduate School of Medicine, Shitsukawa, Toon, Ehime 791-0295 Japan

**Keywords:** Preiser disease, Peadiatric case report, Scaphoid ossification

## Abstract

**Background:**

The pathology of Preiser disease remains controversial, and treatment for Preiser disease has not yet been standardised. Preiser disease itself is rare, and although it can be found in children, its presentation is even rarer; therefore, the treatment of paediatric patients with Preiser disease is more unclear than adult cases.

**Case presentation:**

A 10-year-old boy who complained of left wrist pain was diagnosed with Preiser disease from osteosclerosis and segmentation on plain radiography and computed tomography, and low signal intensity on both T1- and T2-weighted images on magnetic resonance imaging. Because the patient was a child whose scaphoid was immature and pre-ossified, we chose a conservative immobilisation treatment with a thumb spica cast followed by an orthosis. After 3 months of immobilisation, the distal pole of the scaphoid showed remodelling. One year after the initial visit, plain radiography showed remodelling of the whole scaphoid, although magnetic resonance T1-weighted image showed that the recovery of intensity change was only observed in the distal pole. Two years after the initial visit, both plain radiography and magnetic resonance imaging showed a normal appearance and 5 years after the initial visit; the scaphoid bone showed normal development.

**Conclusions:**

This is the first case report of Preiser disease before complete ossification of the scaphoid; therefore, we cannot say anything definitive about the treatment strategy. However, our experience suggests that conservative treatment may provide a cure for Preiser disease in children with immature ossification of the scaphoid without carpal collapse.

## Background

Preiser disease is characterised by avascular necrosis of the scaphoid [1]. The pathology is controversial [2], although, patients typically complain of wrist pain and limitation of wrist motion. Plain radiographs showed scaphoid sclerosis without visible fracture, and magnetic resonance imaging (MRI) demonstrated abnormal intensity in the scaphoid [1]. Many case reports have been recorded since Georg Preiser described this idiopathic osteonecrosis of the scaphoid in 1910 [1]. However, there are few single case series large enough to reveal the aetiology and pathogenesis. Therefore, the treatment of Preiser disease has not yet been standardised. Though it is rare in the first two decades of life [3], this condition can present itself in children [4]; therefore, the therapeutic approach for Preiser disease in children is more unclear than adult cases. Herein, we present a case of Preiser disease in a child before complete ossification of the scaphoid, which was successfully treated by immobilisation.

## Case presentation

**Fig. 1:**
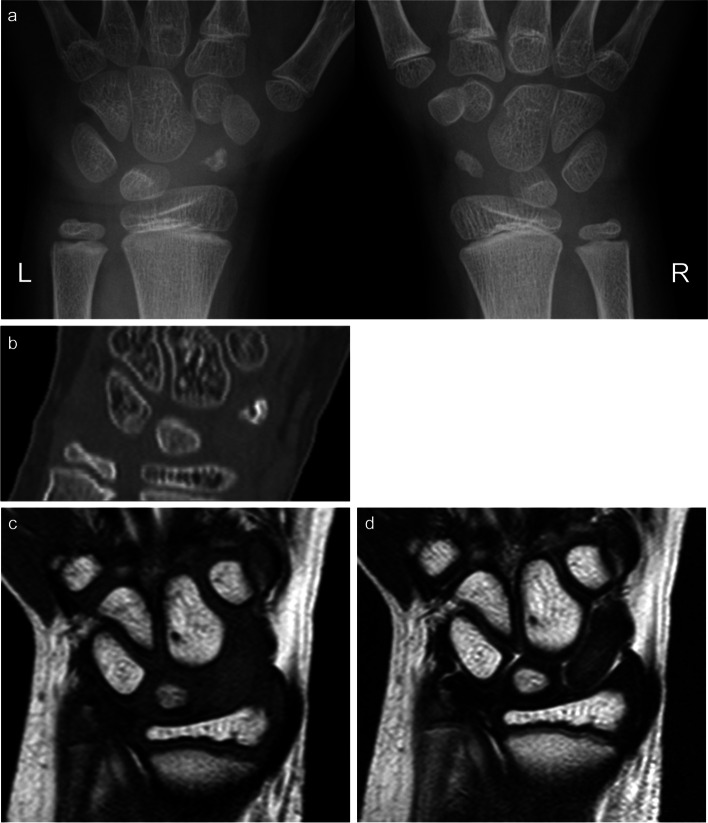
Radiologic image findings at the initial visit. Plain radiography showed osteosclerosis (**a**) and CT revealed segmentation of left scaphoid bone (**b**). Both MRI T1- and MRI T2-weighted images showed low intensity on the whole scaphoid bone, which meant osteonecrosis (**c**, **d**)

A 10-year-old boy complaining of left wrist pain was referred to our hospital. He was in a judo class. He fell off his bicycle and hurt his left wrist 4 days prior to the presentation. He had no remarkable history, familial history, or risk for avascular necrosis of bone. During the physical examination, he reported tenderness in the left anatomical snuff box. The range of motion of the left wrist was not disturbed in comparison to the sound side. The Japanese Society for Surgery of the Hand Version of Quick Disabilities of Arm, Shoulder, and Hand ﻿(QuickDASH-JSSH) disability/symptom score [5, 6] was 6.82. Plain radiography and computed tomography (CT) showed that his scaphoid had not yet undergone complete ossification (Fig. 1a, b), and revealed osteosclerosis, segmentation, and collapse of the ossification nucleus of the left scaphoid. MRI demonstrated an abnormal intensity of the left scaphoid, which showed low intensity on both T1- and T2-weighted images (WI). In addition, there was no oedematous change or bleeding findings (Fig. 1c, d). Firstly, a scaphoid fracture was suspected because the patient complained of wrist pain following the wrist injury. However, plain radiography, CT, and MRI findings were not compatible with scaphoid fractures but with avascular necrosis of the scaphoid. Finally, the patient was diagnosed with idiopathic necrosis of the scaphoid.

**Fig. 2:**
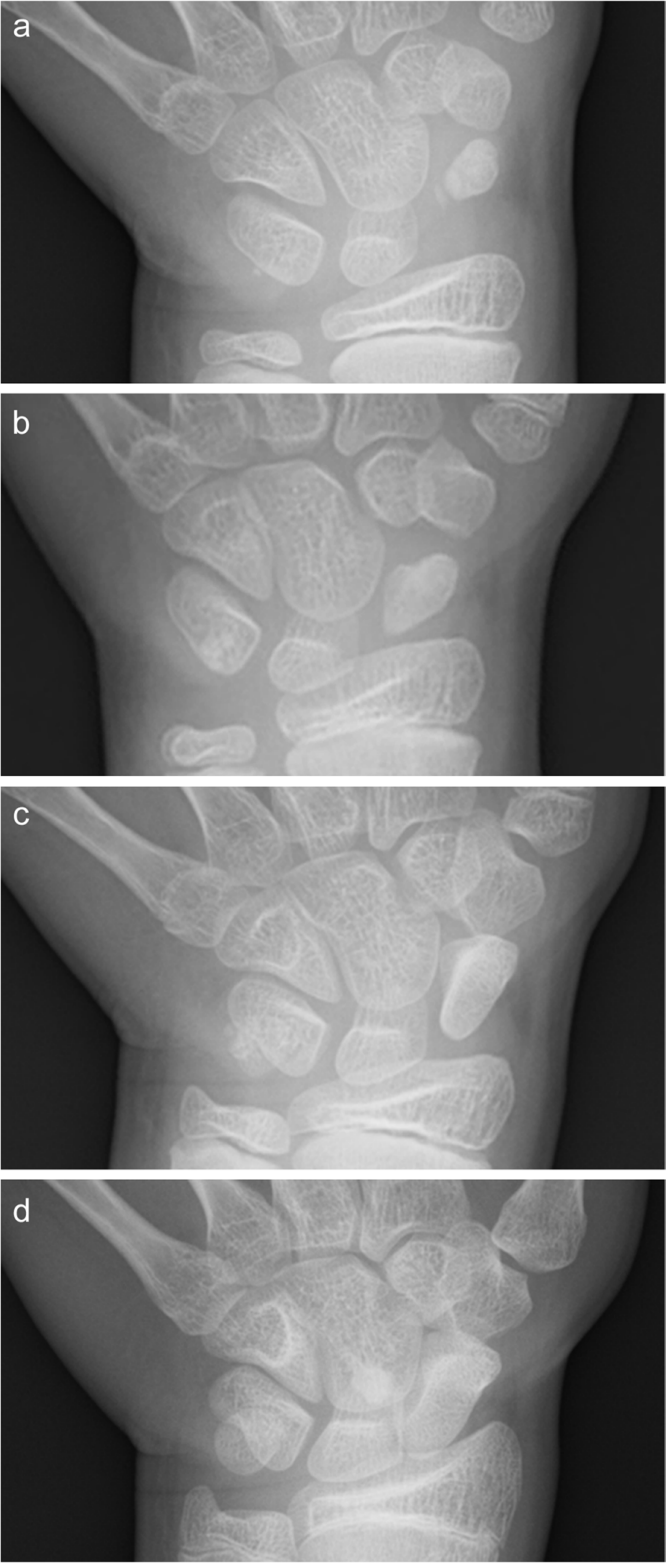
Temporal changes in plain radiography. After 2 month of orthosis immobilisation followed by 3 weeks of immobilisation using a thumb spica cast, the distal pole of the scaphoid showed remodelling (**a**). At 1 year after the initial visit, plain radiography showed remodelling of the proximal pole (**b**). At 2 years after the initial visit, the whole scaphoid bone showed remodelling (**c**). At 5 years after the initial visit, scaphoid bone showed normal growth (**d**)

Although the ossific nucleus showed segmentation and collapse, the patient was young, thus, conservative treatment was performed. A thumb spica cast was applied for 3 weeks, followed by orthosis immobilisation for 2 months. The orthosis was fixed between thumb and forearm, i.e., metacarpophalangeal joint and wrist joints, and it was applied day and night except for bathing. On plain radiography, the segmentation of the ossification nucleus of the left scaphoid disappeared after 3 weeks of cast fixation. The distal pole of the scaphoid showed remodelling, and the laterality of the size of the ossification nucleus of the scaphoid disappeared after 2 months of orthosis immobilisation, following thumb spica casting (Fig. 2a). Also, the tenderness of the anatomical snuff box disappeared during the same period.

**Fig. 3:**
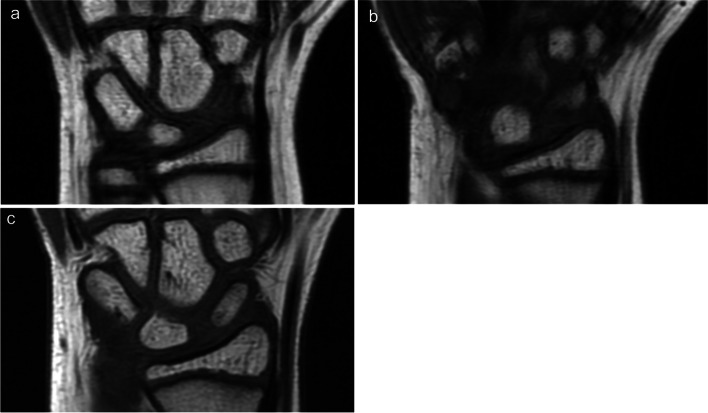
Temporal changes in MRI T1-weighted images. At 1 year after the initial visit, scaphoid bone still showed low intensity (**a**), though partial recovery of intensity change was observed only in the distal pole (**b**). At 2 years after the initial visit, normal intensity on the whole scaphoid bone was observed (**c**)

One year after the first visit, plain radiography showed remodelling of the proximal and distal poles (Fig. 2b). However, MRI demonstrated that bone marrow intensity in the distal pole of the scaphoid was normalised, although the low-intensity area on the proximal pole remained (Fig. 3a, b). Two years after the first visit, plain radiography showed remodelling of the whole scaphoid bone (Fig. 2c), and MRI showed normalisation of bone marrow intensity (Fig. 3c). At the last follow-up, 5 years after the first visit, plain radiography showed normal development of the scaphoid (Fig. 2d), and QuickDASH-JSSH disability/symptom score was 0.

## Discussion and conclusions

Preiser reported cases of rarifying osteitis of the scaphoid that were distinguished from a scaphoid fracture and suggested post-traumatic avascular necrosis [1, 2, 4]; after that, the condition was called Preiser disease [2]. Although Preiser’s original cases were questioned as they show a fracture and no sign of avascular necrosis [2], Preiser disease is still usually defined as post-traumatic avascular necrosis of the scaphoid without fracture. In this report, we also refer to post-traumatic avascular necrosis of the scaphoid as a Preiser disease.

Diagnosis and staging of Preiser disease are made using radiographic images. According to the classification by Herbert and Lanzetta, in the early stage, the radiological examination is normal (stage 1), and next, the ischaemic proximal pole appears relatively dense (stage 2), followed by collapse and cystic change (stage 3), and finally, progressive carpal collapse occurs (stage 4) [7]. However, in early cases, plain radiographs are not sensitive to enough to detect avascular necrosis. MRI with or without gadolinium administration is generally assumed to be the most sensitive modality to assess the vascularity of the scaphoid [8]. Classification based on MRI findings by Kalainov divided Preiser disease into two groups: diffuse ischaemia and necrosis of the scaphoid (type 1) and vascular changes present in only a section of bone (type 2) [9]. The findings of plain radiography and MRI in this report correspond to stage 3 of Herbert and Lanzetta and type 1 of Kalainov. There have been a few case reports of Preiser disease in children; however, none of them occurred in the scaphoid with immature ossification, like in this case. Therefore, radiographic images of this patient could not be compared with those of other cases. However, osteosclerosis and segmentation of the scaphoid in plain radiography and CT [1], and low intensity on both T1-WI and T2-WI on MRI [8, 10] were consistent with some radiographic feature of avascular necrosis of the scaphoid; therefore we diagnosed the patient with Preiser disease.

Various case reports and case series have been reported; however, therapeutic strategies for Preiser disease have not yet been standardised. Although a few reports have recommended conservative treatment for the early stage of Preiser disease [7, 9], recent reports have reported that immobilisation could not be indicated for adults Preiser disease [3, 11]. Surgical debridement [12], denervation [13], localised cortisone injection [9], closing radial wedge osteotomy [14], and vascularised or non-vascularised bone grafts [9, 15] have also been reported. Among them, vascularised bone grafting seems reliable for patients with stage 2 or 3 diseases [15–17]. For more aggressive cases, proximal row carpectomy or partial fusion, for example, four-corner fusion, was performed [3, 13].

The treatment for Preiser disease in children is unclear. We found four cases of Preiser disease in children [18–21]. One patient treated with a vascularised bone graft showed improvement in both clinically and radiologically [21]. Two patients treated with immobilisation showed a good clinical course [18, 20], and the last patient had no mention of the treatment [19]. The patients, in this case, were classified as stage 3 of Herbert and Lanzetta - recommended for vascularised bone grafting, and type 1 of Kalainov, which trends towards scaphoid collapse in adults. We chose conservative treatment because the patient was a child whose scaphoid was immature and pre-ossified. Lenoir reported that conservative treatment was only indicated in paediatric patients with spontaneous revascularisation [3]. Bergman also proposed that conservative treatment should be reserved for young patients, those with pauci-symptoms, and those at an early stage [22]. Since this is the first case report of Preiser disease before complete ossification of the scaphoid, we cannot say anything definitive about the treatment strategy. However, our experience suggests that conservative treatment may provide a cure for Preiser disease in children with immature ossification of the scaphoid without carpal collapse. Establishing a therapeutic strategy for Preiser disease in children requires the observation of even more cases.

## Data Availability

The datasets used and/or analysed during the current study are available from the corresponding author on reasonable request.

## Abbreviations

MRI: Magnetic resonance imaging
QuickDASH-JSSH: The Japanese Society for Surgery of the Hand Version of Quick Disabilities of Arm, Shoulder, and Hand
CT: Computed tomography
WI: weighted images
